# Low Phenotypic Penetrance and Technological Impact of Yeast [*GAR*^+^] Prion-Like Elements on Winemaking

**DOI:** 10.3389/fmicb.2018.03311

**Published:** 2019-01-09

**Authors:** Ramon Gonzalez, Jordi Tronchoni, Ana Mencher, José Antonio Curiel, Alda Joao Rodrigues, Laura López-Berges, Cristina Juez, Kiran Raosaheb Patil, Paula Jouhten, Noelia Gallego, Alejandra Omarini, Mariana Fernández-Preisegger, Pilar Morales

**Affiliations:** ^1^Instituto de Ciencias de la Vid y del Vino (CSIC, Universidad de La Rioja, Gobierno de La Rioja), Logroño, Spain; ^2^Structural and Computational Biology Unit, European Molecular Biology Laboratory, Heidelberg, Germany; ^3^Industrial Biotechnology and Food Solutions, VTT Technical Research Centre of Finland Ltd., Espoo, Finland; ^4^INCITAP Consejo Nacional de Investigaciones Científicas y Técnicas – Universidad Nacional de La Pampa, Santa Rosa, Argentina; ^5^Consejo Nacional de Investigaciones Científicas y Técnicas – UNER Universidad Nacional de Entre Ríos – Centro de Investigaciones Científicas y Transferencia de Tecnología a la Produccion, Diamante, Argentina

**Keywords:** wine yeast, prion-like, phenotypic penetrance, ethanol yield, volatile acidity, aerobic fermentation

## Abstract

[*GAR*^+^] prion-like elements partially relieve carbon catabolite repression in *Saccharomyces cerevisiae*. They have been hypothesized to contribute to wine yeast survival and alcohol level reduction, as well as communication with bacteria and stuck fermentation. In this work, we selected [*GAR*^+^] derivatives from several genetic backgrounds. They were characterized for phenotypic penetrance, heritability and confirmed as prion-like through curing by desiccation. In terms of fermentation kinetics, the impact of the prion on anaerobic wine fermentation (natural grape juice) was either neutral or negative, depending on the genetic background. Likewise, residual sugars were higher or similar for [*GAR*^+^] as compared to the cognate [*gar*^-^] strains. The prions had little or no impact on glycerol and ethanol yields; while acetic acid yields experienced the highest variations between [*GAR*^+^] and [*gar*^-^] strains. Strains analyzed under aerobic conditions followed the same pattern, with either little or no impact on fermentation kinetics, ethanol or glycerol yield; and a clearer influence on volatile acidity. Although no clear winemaking advantages were found for [*GAR*^+^] strains in this work, they might eventually show interest for some combinations of genetic background or winemaking conditions, e.g., for reducing acetic acid yield under aerated fermentation.

## Introduction

*Saccharomyces cerevisiae* is the main yeast species responsible for alcoholic fermentation of many traditional foods and beverages. It exhibits alcoholic fermentation even under aerobic conditions, making it an archetypical Crabtree-positive yeast. The Crabtree effect is characterized by low alcoholic fermentation until sugars are almost exhausted, although oxygen availability would allow for more efficient ATP production via respiration. In contrast, Crabtree-negative yeasts show respiratory metabolism under these conditions, producing almost exclusively carbon dioxide and biomass, and no ethanol. Several biological mechanisms contribute to the Crabtree effect, including carbon catabolite repression (CCR) of mitochondrial and respiration related genes (Schüller, 2003; Kayikci and Nielsen, 2015).

Carbon catabolite repression is a common metabolic feature of unicellular microorganisms, allowing them to adapt to changing levels of nutrient availability by selecting the most favorable carbon source when exposed to mixtures. Evolution has shaped different CCR mechanisms in different phyla of Bacteria and Eukarya, operating in each case by several overlapping mechanisms (Gancedo, 2008; Görke and Stülke, 2008). The paradigm of CCR being clearly advantageous for microbial species was somewhat challenged by the discoveries around the [*GAR*^+^] prion-like element in *S. cerevisiae*. [*GAR*^+^] is a protein-based heritable element that allows yeast circumvent CCR, so becoming a metabolic generalist, in contrast to the specialization for glucose shown by wild-type strains (Brown and Lindquist, 2009). The capability of relieving glucose repression has been proposed as an evolutionarily conserved mechanism of bet-hedging in fluctuating environments, providing some adaptive advantage, e.g., rapid reversibility, over DNA mutation (Jarosz et al., 2014b).

Some features described for *S. cerevisiae* strains carrying the [*GAR*^+^] determinant, are potentially attractive from an oenological perspective. Notably, Jarosz et al. (2014a), reported a reduction from 14% alcohol strength to 12% in the fermentation of a white grape juice. In addition, they found that some bacterial strains induce *de novo* appearance of [*GAR*^+^] and analyzed the potential mutual advantages for each partner (yeast or bacteria) of [*GAR*^+^] induction in an evolutionary and ecological context. The nature of the inducing molecule is still controversial, since some authors found lactic acid (Garcia et al., 2016) as the main responsible metabolite, while others considered it was acetic acid, finding no effect of lactic acid on prion induction (Ramakrishnan et al., 2016).

These findings prompted us to investigate whether this epigenetic element might be advantageous in reducing alcohol content of wines. Excess alcohol content is a problem, related to global climate change, specially affecting winemaking in warm climate countries (Mira de Orduña, 2010). Our research group has proposed using the diversity of yeast metabolism to reduce ethanol yield during fermentation (Gonzalez et al., 2013; Ciani et al., 2016). Our strategy involves carbon redirection to respiratory metabolism in *S. cerevisiae*, and using alternative wine yeast species (Quirós et al., 2014; Morales et al., 2015). We also identified acetic acid production under aerobic conditions as a major drawback of respiration by *S. cerevisiae* in grape must (Curiel et al., 2016). However, the results by Jarosz et al. (2014a) suggested using the prion state of *S. cerevisiae* could be an additional strategy. In this work we analyzed the effect of [*GAR*^+^] prion elements on fermentation kinetics and on the yield of main fermentation metabolites in natural grape must. We investigated the effects under both aerobic and anaerobic conditions and using six different wine yeast strain backgrounds. Our findings do not support a practical usage of the prion state based on lower ethanol yield, since no appreciable impact was found. However, a potentially interesting impact on aerobic acetic acid production was identified for some genetic backgrounds.

## Materials and Methods

### Strains and Media (“Selective” and “Permissive”)

Six *S. cerevisiae* wine yeast strains were used for prion recovery in this work. FX10 (Laffort), EC1118 (Lallemand Inc.), T73 (Lallemand Inc.), and UCD522 (maurivin) are commercial winemaking strains. IFI87 and IFI473 are strains from the collection of the ancient Instituto de Fermentaciones Industriales (Madrid, Spain) and are currently deposited at the CECT collection as, respectively, CECT12512 and CECT12658. In addition, a [*GAR*^+^] derivative of the laboratory strain W3031A, together with its [*gar*^-^] counterpart, kindly provided by Daniel F. Jarosz, were used for reference purposes. Strains were routinely maintained on YPD plates (1% yeast extract, 2% peptone, 2% glucose, 1.5% agar), or as glycerol stocks (20% glycerol) at -80°C. GGM (1% yeast extract, 2% peptone, 2% glycerol, 0.05% glucosamine, 1.5% agar) was used as selective medium for prion isolation and phenotypic confirmation. YPGly (1% yeast extract, 2% peptone, 2% glycerol, 1.5% agar) was used as permissive medium for the growth of both [*gar*^-^] and [*GAR*^+^] strains. Ingredients for culture media were of at least 99% purity, purchased from Sigma-Aldrich. Agar was purchased from Thermo Fisher (Oxoid).

### Fermentation Experiments

Fermentation experiments were run in either Falcon tubes or MiniBio Bioreactors (Applikon Biotechnology B.V., Delft, Netherlands). Pre-cultures were grown in YPD (1% yeast extract, 2% peptone, 2% glucose) for 48 h at 25°C. All experiments were run in triplicate and three independent inocula were prepared for each strain and culture condition. Fermentation medium was a natural white grape must (*Vitis vinifera* L. cv. Viura) from the 2017 harvest (Finca La Grajera, La Rioja, Spain), containing about 100 g/L glucose, 98 g/L fructose, pH 3.6. It was stored in frozen aliquots (-20°C). Before use, in order to reduce microbial load, it was thawed and submitted to thermal treatment by heating in an autoclave until reaching 105°C; and then, immediately, stopping heating and allowing the autoclave to cool down.

Yeast cells were grown on YPD for 48 h at 25°C; then washed with the same volume of distilled water and used as inocula for fermentation assays. Small volume fermentations, inoculated at a final OD_600_ of 0.2, were run in Falcon tubes (50 mL nominal volume) containing 20 mL of grape must and closed with fermentation locks (filled with mineral oil). Fermentation kinetics was monitored by daily recording weight loss. Fermentations were incubated at 25°C (static) and stopped after 10 days. By the end of the experiment, cultures were centrifuged and fermentation metabolites in the supernatant analyzed by HPLC.

Fermentations in laboratory scale bioreactors (250 mL nominal volume), were performed with the same batch of natural grape must as above, with 150 mL working volume. Cultures were inoculated at a final OD_600_ of 0.2 (with inocula prepared as described above). They were sparged with dry air or nitrogen, for aerobic or anaerobic conditions, respectively. Flow of the input gas was adjusted to 25 mL/min with MFC17 mass flow controllers (Aalborg Instruments and Controls, Inc., Orangeburg, NY, United States) whose calibration was regularly verified with an electronic flowmeter (Agilent Technologies, Santa Clara, CA, United States). CO_2_ content in the output gas flow was recorded every 60 s with the aid of BlueInOne Cell gas analysers (BlueSens, Germany) connected to each bioreactor. Stirrer was set at 1000 rpm and temperature at 25°C.

### Analytical Methods

Concentration of the main metabolites, glucose, fructose, glycerol, acetic acid and ethanol, was determined in duplicate for each sample, by HPLC, using a Surveyor Plus chromatograph (Thermo Fisher Scientific, Waltham, MA, United States) equipped with a refraction index and a photodiode array detector (Surveyor RI Plus and Surveyor PDA Plus, respectively). HI-Plex H 300 mm × 7.7 mm column and guard (Agilent Technologies) were used and maintained at 50°C. Elution was performed with 1.5 mM H_2_SO_4_ as mobile phase, at 0.6 mL/min. Prior to injection, samples were filtered through 0.22-μm-pore-size nylon filters and diluted 10-fold.

#### Isolation of [*GAR*^+^] Colonies and Confirmation of Derepressed Phenotype

Putative [*GAR*^+^] strains were recovered according to Brown and Lindquist (2009). Briefly, yeast cells were grown in YPD for 16 h at 25°C and 150 rpm, and suitable dilutions plated on GGM selective medium. Colonies arising after 3–10 days, depending on the genetic background, were streaked on GGM to obtain pure cultures. The derepressed phenotype was then confirmed by serial drop tests on GGM. To this end, precultures were grown on YPD for 48 h, cells were washed in distilled water and adjusted to 1 unit of OD_600_. Serial (1/10) dilutions were prepared on 96-well microplates, with a multichannel automatic pipette, and used to inoculate rectangular agar plates (GGM) with a manual 96-pin replica plater. Control plates were inoculated in parallel from the same yeast cell dilutions.

#### Phenotypic Penetrance, Heritability and Desiccation Assays

Phenotypic penetrance and heritability were studied in association with the analysis of the impact of desiccation. The assay was based on the method described by Tapia and Koshland (2014). [*GAR*^+^] cells were grown on YPD until constant OD (about 4 days). The cultures were washed on the same volume of diluted PBS (1:80) and distributed in screw cap microcentrifuge tubes (2 mL per tube), centrifuged again and the supernatant carefully removed. For initial values of penetrance and heritability, this pellet was immediately suspended in water and dilutions prepared for further analysis. For the impact of desiccation, pellets were incubated at 25°C for up to 10 weeks. For each time point one tube from each strain was suspended in water and used for penetrance and heritability quantification.

Penetrance of prions on each genetic background was quantified as the ratio between colony counts for each [*GAR*^+^] strain in selective versus permissive medium (GGM vs. YPGly). These plates were inoculated with suitable dilutions of the cell suspensions prepared as described above, and plates containing 50–300 colonies counted after 3–7 days, depending on the genetic background and the culture medium. For the quantification of heritability, 10 single colonies from permissive medium were checked for prion status as described in the previous section. Heritability was calculated as the fraction of colonies retaining the [*GAR*^+^] phenotype.

### Strain Background Confirmation

In order to rule out any contamination event as the explanation of the results obtained in the phenotypic characterization, desiccation, penetrance, and heritability assays, strain background was routinely checked for the different isolates by interdelta analysis. DNA was extracted as described by Lõoke et al. (2011). Amplification and electrophoresis of interdelta elements was done as described by Legras and Karst (2003).

### Statistical Analysis

Yields of the main fermentation metabolites were compared by one-way analysis of variance. Correlation between phenotypic penetrance and time of desiccation was based on the Pearson correlation coefficient. All analyses were performed using SPSS Statistics v. 25 program (IBM, Armonk, NY, United States).

## Results

### Penetrance and Heritability of [*GAR*^+^] Phenotype

In order to study the relevance of [*GAR*^+^] on winemaking the first step was to obtain wine yeast strains carrying this prion-like element. Six different genetic backgrounds (as well as W303 strain as a reference) were used for this purpose. [*GAR*^+^] strains were identified as described in Section “Materials and Methods” and by Brown and Lindquist (2009). Colonies growing on selective medium (i.e., able to use glycerol in the presence of glucosamine) appeared at rates ranging from below 0.01 to around 1% (Table 1). The rate obtained for W303 was in the order of magnitude described by Brown and Lindquist (2009). One colony growing on selective medium from each genetic background was retained as potential [*GAR*^+^] derivative. Most of them were also able to use raffinose or maltose in the presence of glucosamine but were not derepressed for the use of glycerol in the presence of 2-deoxy-glucose (data not shown). This indicates the CCR loosening under the prion state being, at most, incomplete with some glucose repression pathways still active.

**Table 1:** Frequency of appearance of [GAR^+^] phenotype in different yeast strains.

Strain	Prion isolation frequency (%)
W303	<0.01
FX10	0.1–0.5
EC1118	0.5–1
UCD522	0.5–2
T73	<0.01
IFI87	0.1–0.5
IFI473	0.1–1


**FIGURE 1:**
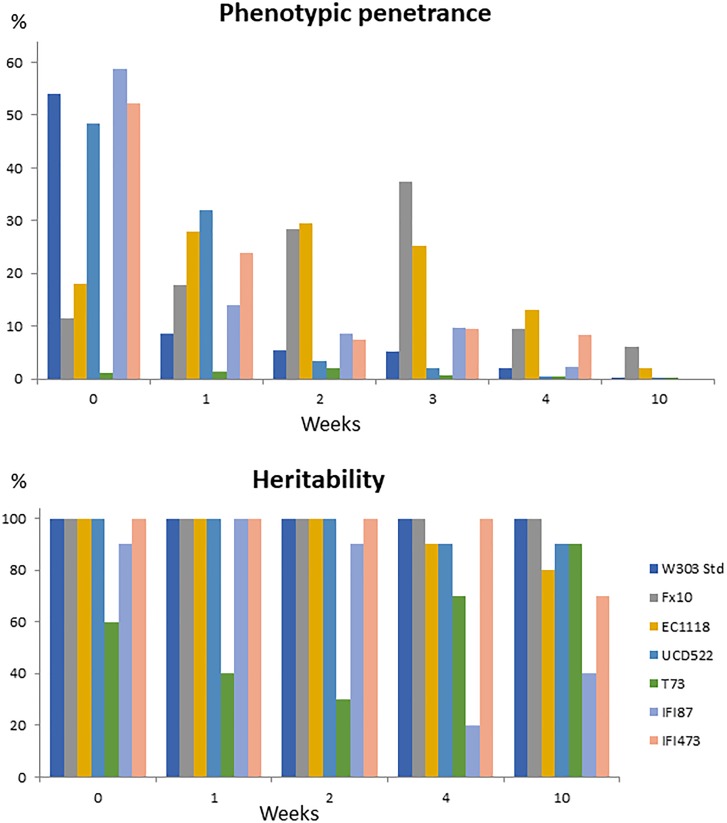
Phenotypic penetrance and heritability of the prion state for different yeast genetic backgrounds, under standard conditions (week 0) or after several weeks under desiccation conditions.

An important feature to consider toward understanding the relevance of [*GAR*^+^] prion-like elements in the wine making process would be phenotypic penetrance. The first trial to confirm the prion state of these strains was based on the expectation that plating [*GAR*^+^] strains in parallel in selective and permissive medium would result in similar colony counts. However, colony counts in selective medium fell far below those in the control medium. Indeed, despite penetrance was not analyzed in the original descriptions of [*GAR*^+^] (Brown and Lindquist, 2009; Jarosz et al., 2014a,b), low phenotypic penetrance had been indirectly reported for this prion-like element (Tapia and Koshland, 2014). We therefore quantified the penetrance for all the [*GAR*^+^] strains under standard conditions (Figure 1, initial time-point). Values ranged from around 50%, for W303-1A[*GAR*^+^] and three of the wine yeast strains, to values below 20% for three of the winemaking strains in prion state (Figure 1). T73[*GAR*^+^] low penetrance, below 2%, was striking. But, low phenotypic penetrance was distinct from the loss of the prion state. Indeed, before desiccation, most [*GAR*^+^] strains showed 100% heritability, and the actual value for T73 was as high as 60% (Figure 1). The low penetrance of T73[*GAR*^+^] on this assay did not preclude the prion to impact yeast physiology under winemaking conditions (see below).

Reversibility (or the ability to be cured) is another key feature of [*GAR*^+^] epigenetic elements distinguishing them from classical, nucleic acid based, genetic determinants (Brown and Lindquist, 2009). Curing by desiccation has been shown, for [*GAR*^+^] strains, by Tapia and Koshland (2014) and Ramakrishnan et al. (2016), the latter authors using it as a diagnostic tool for the prion state. However, their experimental setup (just based on the ratio of colony counts between selective and permissive media), did not allow to distinguish between phenotypic penetrance or heritability drop by desiccation. In this work, both features have been assessed separately by performing a phenotypic analysis of colonies randomly picked from permissive medium plates, to assess heritability. Although the behavior during the 1st weeks tended to be random, probably due to limitations on the experimental setup, a clear penetrance drop was observed for all the [*GAR*^+^] strains tested for prolonged desiccation periods, (Figure 1), with five out of seven strains showing 0% phenotypic penetrance by the 10th week. The Pearson correlation coefficient for phenotypic penetrance against desiccation time was -0.528, with a signification <0.001. In contrast, full heritability was maintained for five of the strains up to the 2nd week (Figure 1), with a steady decrease afterwards, until the end of the experiment (10th week). By that time, all but one of the [*GAR*^+^] strains deriving from wine yeasts showed some colonies were the prion state had been lost. Interdelta analysis was used to rule out contamination as the origin of the [*gar*^-^] strains isolated in the desiccation assays. The random pattern of heritability shown by strain T73[*GAR*^+^] might also be related to the low penetrance of the prion on this genetic background.

### Impact on Fermentation Kinetics and Yields of the Main Fermentation Metabolites

**FIGURE 2:**
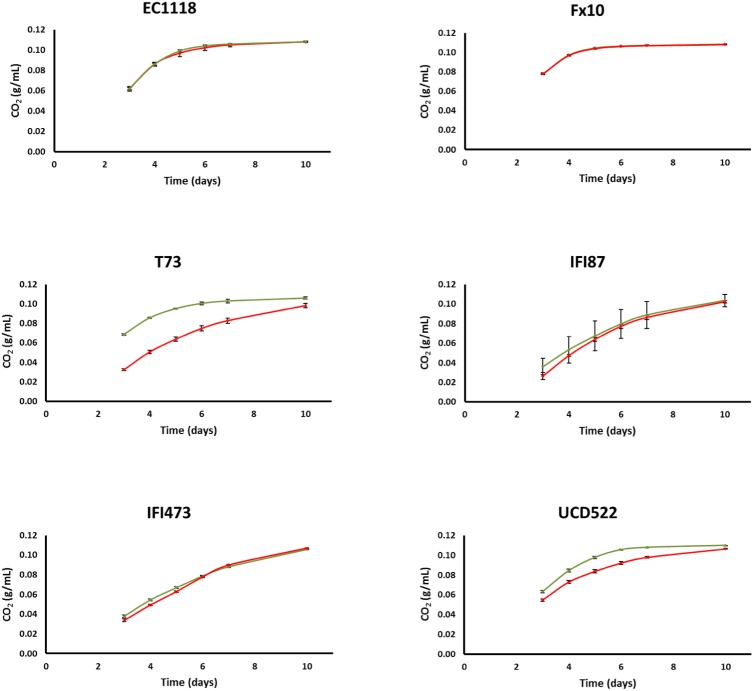
CO_2_ released by different wine yeast strains during the fermentation of natural grape must (estimated by weight loss). The genetic background is indicated in each panel. Values for the [*gar*^-^] and [*GAR*^+^] phenotypes are shown in green and red color, respectively. Error bars indicate ±SD from three biological replicates.

Fermentation kinetics on natural grape must was evaluated in small volumes (20 mL), comparing the [*gar*^-^] and [*GAR*^+^] strains of each one of the six industrial genetic backgrounds. In three cases, viz., EC1118, FX10, and IFI473, the fermentation profiles of the original strains and those harboring the prion were indistinguishable (Figure 2). For the other three genetic backgrounds, [*GAR*^+^] strains ranged from slightly (IFI87) to clearly impaired (T73 and UCD522) fermentation kinetics; with weight losses for the prion strain 30% or 115% below the control strain by the 5th day of fermentation. It is worth noting the strong effect of the prion state in the T73 background despite the low penetrance described above. This would indicate that phenotypic penetrance on the plate assay is not fully related with the prion behavior under different culture conditions. In agreement with these fermentation profiles, residual sugar by the end of fermentation was either similar or increased for most strains under the prion state (Supplementary Table S1), with the only exception of IFI473[*GAR*^+^]. The increase in residual sugars (mostly fructose) was especially striking in the T73 background (Supplementary Table S1); increasing from 4 g/L for T73[*gar*^-^], to almost 20 g/L for T73[*GAR*^+^]. Importantly, ethanol yields were similar for all the strains, either [*gar*^-^] or [*GAR*^+^], by the end of fermentation (Supplementary Table S2), in line with findings published recently by Walker et al. (2016). In most cases, differences on glycerol yields between [*gar*^-^] and [*GAR*^+^] strains were statistically significant (Supplementary Table S2). However, the dimension of the change was small, and there was no clear trend with respect to the direction of the change; i.e., glycerol content increased in some genetic backgrounds and decreased in others (Supplementary Table S1). Similarly, acetic acid yield was clearly affected by the prion status. The impact was null for two strains, while three of them showed reduced yield, and it was increased for UCD522 (Supplementary Table S2). In contrast to glycerol or ethanol yields, in most cases this resulted in big changes on final acetic acid concentration in wines, which ranged from 0.5 to 1.5 times, for the [*GAR*^+^] strains, as compared to the [*gar*^-^] counterpart (Supplementary Table S1).

### Differential Impact Under Aerobic or Anaerobic Conditions

Two strain backgrounds, FX10 and UCD522, were selected to further characterize their metabolism under winemaking conditions. The first one was representative of the strains whose behavior was mostly unaffected by the prion status in the previous experiments, while the second one was among those more clearly impaired by [*GAR*^+^]. Considering previous reports that relate the [*GAR*^+^] epigenetic element with reduced ethanol yield (Jarosz et al., 2014b) and eventually higher respiration rate, we performed fermentation experiments in bioreactors under both aerobic and anaerobic conditions.

**FIGURE 3:**
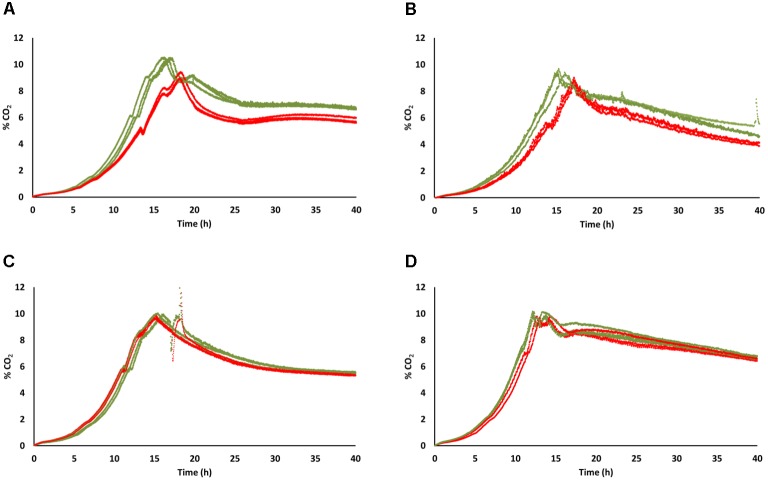
Rate of CO_2_ release during the first 40 h of fermentation of natural grape must in bioreactors. Three experimental replicates are shown for each strain and condition, with data for the [*gar*^-^] and [*GAR*^+^] phenotypes shown in green and red color, respectively. **(A)** UCD522 under aerobic conditions. **(B)** UCD522 under anaerobic conditions. **(C)** FX10 under aerobic conditions. **(D)** FX10 under anaerobic conditions.

Anaerobic fermentation profiles, recorded for the first 40 h of cultivation, confirmed the trend observed in lower volumes in cultivation tubes. [*GAR*^+^] and [*gar*^-^] profiles were almost overlapping for the FX10 background, while UCD522[*GAR*^+^] CO_2_ production was below that of the original strain, with the maximum being reached around 2 h later (Figure 3). Under aerobic conditions CO_2_ production profiles followed a similar trend, with FX10[*GAR*^+^] closely resembling the original FX10 strain, while UCD522[*GAR*^+^] showed a delayed and less vigorous fermentative behavior than its [*gar*^-^] counterpart; reaching maximum CO_2_ production rate about 10% below and about 2 h later (Figure 3). These differences on CO_2_ production patterns of UCD522[*GAR*^+^] and UCD522[*gar*^-^] were more apparent under aerobic than under anaerobic conditions (Figure 3).

Sugar consumption profiles during the first 2 days of fermentation faithfully mirrored the trends observed for CO_2_ production. They were mostly unaffected by the prion state in the FX10 background. While the impact of the prion in the UCD522 background was higher for aerobic than for anaerobic conditions (Supplementary Figure S1). Accordingly, residual sugar after fermentation arrest under aerobic conditions was much higher for UCD522[*GAR*^+^] than for UCD522[*gar*^-^] (70.8 and 43.5 g/L, respectively). Aerobic cultures on the FX10 background were also arrested before complete sugar consumption, but the difference between both strains (prion or not) was almost negligible.

**FIGURE 4:**
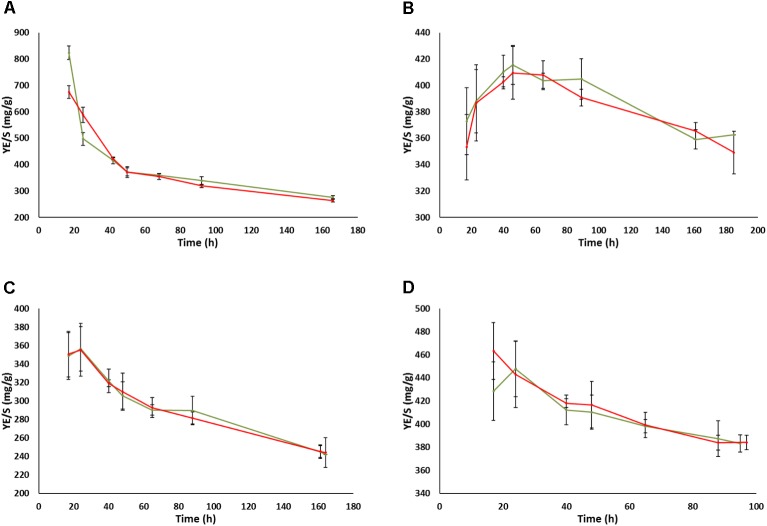
Evolution of ethanol yields during the fermentation of natural grape must in bioreactors. **(A)** UCD522 under aerobic conditions. **(B)** UCD522 under anaerobic conditions. **(C)** FX10 under aerobic conditions. **(D)** FX10 under anaerobic conditions. Data for the [*gar*^-^] and [*GAR*^+^] phenotypes are shown in green and red color, respectively. Error bars indicate ±SD from three biological replicates.

**FIGURE 5:**
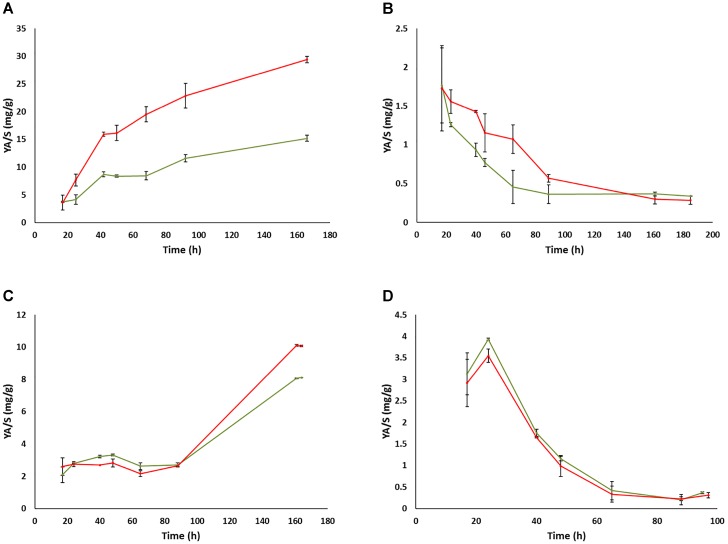
Evolution of acetic acid yields during the fermentation of natural grape must in bioreactors. **(A)** UCD522 under aerobic conditions. **(B)** UCD522 under anaerobic conditions. **(C)** FX10 under aerobic conditions. **(D)** FX10 under anaerobic conditions. Data for the [*gar*^-^] and [*GAR*^+^] phenotypes are shown in green and red color, respectively. Error bars indicate ± SD from three biological replicates.

In contrast to the impact of prion-like elements on the fermentation kinetics and residual sugars in the UCD522 background, almost no impact of the prion-like element was observed concerning ethanol (Figure 4) or glycerol (Supplementary Figure S2) total yields in the end of fermentations, neither for FX10 nor for UCD522, and neither under aerobic nor under anaerobic culture conditions. This is in agreement with the results described above for small volume fermentation experiments in the final sample point. In contrast, acetic acid yields showed a clear impact of the [*GAR*^+^] element along the fermentation time (Figure 5 and Supplementary Table S3). This impact was again more pronounced in the UCD522 than in the FX10 background (100% vs. 20% increase under aerobic conditions), and also stronger under aerobic than under anaerobic conditions (no statistically significant difference was observed for the later). Indeed, the effect of [*GAR*^+^] on acetic acid yield was completely lost by the end of anaerobic fermentations of the UCD522 background. For aerobic cultures the difference between [*gar*^-^] and [*GAR*^+^] strains increased over time. Acetic acid yield was lower for FX10 strains than for UCD522. For FX10 strains, an increase in acetic acid production was observed by the end of the fermentation, which was somewhat higher for the [*GAR*^+^] strain, increasing from about 8 mg/g to 10 mg/g (Supplementary Table S3). On the contrary, UCD522 produced high amounts of acetic acid from almost the 1st day of fermentation. The values were higher for UCD522[*GAR*^+^] until the fermentation arrest. In this final sample point the acetic acid yield was almost twice for UCD522[*GAR*^+^] (30 mg/g) than for its [*gar*^-^] counterpart (15 mg/g) (Figure 5 and Supplementary Table S3).

## Discussion

In this work, we describe the impact of prion-like [*GAR*^+^] elements on the fermentative behavior and the yield of main fermentation metabolites for six different wine yeast strains. Strains carrying this epigenetic element were selected as described by Brown and Lindquist (2009). Several features confirm they are *bona fide* [*GAR*^+^] strains, apart from the ability to readily grow on GGM plates (i.e., containing glucosamine, as CCR trigger, and glycerol as carbon source). These include, frequency of isolation [on the range described for other wine yeast strains (Jarosz et al., 2014b) and far above mutational frequency], high heritability, penetrance values clearly below 100%, reversion and decreasing penetrance induced by conditions affecting protein folding, like desiccation as described by Tapia and Koshland (2014). Low phenotypic penetrance of [*GAR*^+^] dependent phenotypes, and its variability between strain backgrounds, were not analyzed in the original descriptions of this prion-like element (Brown and Lindquist, 2009; Jarosz et al., 2014a,b) but were later shown by Tapia and Koshland (2014) and also mentioned by Ramakrishnan et al. (2016). It should be stressed that, ignoring the low phenotypic penetrance might complicate the recognition of prion status. In addition, this low penetrance means that circa 50–80% of the [*GAR*^+^] cells might not actually show the associated phenotype. Finally, we observed that [*GAR*^+^] prions relieve only one part of the complex glucose repression system of *S. cerevisiae*. This is illustrated by the effect of 2-deoxyglucose, inhibiting growth of all the [*GAR*^+^] strains assayed on glycerol. This is an important consideration for the potential technological impact of these epigenetic elements.

Concerning fermentation performance on natural grape must, induction of the prion conferred no advantage to wine yeast strains. It was rather the opposite for some of the strains tested. This is in accordance with the results by Walker et al. (2016) who, for the UCD932 strain background, found some negative impact of [*GAR*^+^] on fermentation kinetics in the presence of SO_2_, that was magnified in natural grape must without SO_2_, due to competition with natural microbiota under their experimental conditions.

One feature of [*GAR*^+^] strains described by Jarosz et al. (2014a), draws special attention because of its potential impact on the final alcohol content of wines. That study reported lower ethanol yield for [*GAR*^+^] strains both in laboratory medium and grape juice, and both for a laboratory and a wine yeast genetic background (W303 and UCD922). However, the work by Walker et al. (2016), also using the UCD922 background, showed a much modest impact of [*GAR*^+^] on ethanol content, and apparently no impact on ethanol yield, since in that case reduced ethanol content was concurrent with a comparable increase in residual sugar. Our results, starting with six different wine yeast strains, point to the same direction as Walker et al. (2016), suggesting that those by Jarosz et al. (2014a) might be specific for some genetic backgrounds, grape juice/wine must composition, and/or fermentation conditions. According to this, the expectation is that inducing the prion state is unlikely to have appreciable effect on ethanol yield in pure *S. cerevisiae* cultures under standard fermentation conditions.

In addition, one strategy to lower ethanol levels in wine that is currently under research is based on respiratory metabolism (Gonzalez et al., 2013; Morales et al., 2015; Curiel et al., 2016; Rodrigues et al., 2016). Since CCR plays also a role on the respiro-fermentative balance of *S. cerevisiae*, the present work went a step forward, analyzing the effect of the prion under aerobic fermentation conditions. However, ethanol yields were again similar for each strain background, regardless of the prion status. There were no indications of changes on the respiro-fermentative balance due to [*GAR*^+^] prions.

Besides ethanol, in this work we quantified glycerol and acetic acid yields. In the range found in wines, glycerol has been often associated to positive sensory properties of wines (Jones et al., 2008; Laguna et al., 2017); while acetic acid, above a certain threshold might result in wine spoilage by contributing to volatile acidity (Ribéreau-Gayon et al., 2006). As with ethanol, glycerol yields were largely unaffected by the [*GAR*^+^] prion, either under aerobic or anaerobic conditions. Acetic acid yield was especially interesting because its production was previously identified as a major drawback when using *S. cerevisiae* to reduce ethanol content by respiration (Morales et al., 2015). It was also previously shown that, some mutant strains, with alleviated CCR, displayed reduced acetic acid production under aerobic fermentation conditions (Curiel et al., 2016). Among the metabolic features of wine yeasts analyzed in this work, this was the most severely affected one. This impact depended on the aeration regime, as well as on the genetic background. For the two strain backgrounds analyzed under aerobic conditions, the one showing the higher impact on fermentation kinetics was also the one showing the higher increase in aerobic acetic acid production. Hence, no technological advantage was found for strains harboring the prion, concerning alcohol level reduction in wine.

## Conclusion

There were two main reasons to anticipate a technological impact of [*GAR*^+^] epigenetic elements in winemaking. On one side, the reports of bacteria commonly found on the winemaking environment, or their metabolites, as inducers of the transition to the prion state, suggested [*GAR*^+^] can be behind some cases of stuck fermentation (Walker et al., 2016). On the other side, lower ethanol production, reported by some authors (Jarosz et al., 2014b), suggests the use of [*GAR*^+^] strains might help reducing alcohol content of wines. However, results described in the present work suggest that the technological relevance of [*GAR*^+^] is likely to be low.

Results on heritance and phenotypic penetrance suggest that, despite they keep the [*GAR*^+^] element, there is always a significant fraction of the population that is not expressing the phenotype. Anaerobic cultures inoculated with pure [*GAR*^+^] strains were either unaffected or delayed, but fermentation was not sluggish or stuck. However, since induction of [*GAR*^+^] after fermentation start would necessarily involve only a fraction, rather than the whole yeast population, prion induction would be expected to have a limited impact on fermentation kinetics.

Considering alcohol level reduction, our results on six different wine yeast genetic backgrounds, do not confirm any significant impact on ethanol yield, neither under aerobic nor under anaerobic conditions. Despite the Crabtree effect, alcohol yields for *S. cerevisiae* are lower under aerobic conditions, as previously described (Quirós et al., 2014). But, increased volatile acidity was one of the major hurdles previously found for using *S. cerevisiae* as part of the respiro-fermentative approach to alcohol level reduction (Morales et al., 2015). A positive impact of the prion state on acetic acid yield (by lowering it) might have provided some advantages for an industrial use of [*GAR*^+^] strains. However, our results were variable, depending on the strain background. Nonetheless, some useful strains for this purpose might eventually arise from a larger screening under aerobic conditions, or by combining [*GAR*^+^] phenotype with other strategies.

## Availability of Data and Material

The data set supporting the results of this article is included in the article (and its Additional files).

## Author Contributions

RG, PM, and KP conceived and designed the study. LL-B, AM, CJ, AR, JC, JT, NG, MF-P, and AO performed the experiments. RG, PM, AM, LL-B, and CJ analyzed the data. RG, KP, PJ, PM, and JT interpreted the results. RG, KP, and PJ wrote the manuscript. All authors discussed and approved the manuscript.

## Conflict of Interest Statement

The authors declare that the research was conducted in the absence of any commercial or financial relationships that could be construed as a potential conflict of interest.

## Supplementary Material

FIGURE S1 Evolution of residual sugar during the fermentation of natural grape must in bioreactors. **(A)** UCD522 under aerobic conditions. **(B)** UCD522 under anaerobic conditions. **(C)** FX10 under aerobic conditions. **(D)** FX10 under anaerobic conditions. Data for the [*gar*^-^] and [*GAR*^+^] phenotypes are shown in green and red color, respectively. Error bars indicate ±SD from three biological replicates.

FIGURE S2 Evolution of glycerol yields during the fermentation of natural grape must in bioreactors. **(A)** UCD522 under aerobic conditions. **(B)** UCD522 under anaerobic conditions. **(C)** FX10 under aerobic conditions. **(D)** FX10 under anaerobic conditions. Data for the [*gar*^-^] and [*GAR*^+^] phenotypes are shown in green and red color, respectively. Error bars indicate ±SD from three biological replicates.

TABLE S1 Final concentrations (10 days) of the main fermentation related metabolites during the fermentation of natural grape must by different yeast strains.

TABLE S2 Yield of the main fermentation metabolites and consumed sugars (10 days) during the fermentation of natural grape must by different yeast strains (Y_E/S_ ethanol yield on sugar, Y_A/S_ acetic acid yield on sugar, Y_G/S_ glycerol yield on sugar). Statistically significant differences for each parameter between the [*gar*^-^] and [*GAR*^+^] with the same genetic background are indicate by ^∗^ (*p*-value < 0.05) or ^∗∗^ (*p*-value < 0.001).

TABLE S3 Final yield of the main fermentation metabolites and consumed sugars after 40 h of fermentation during the fermentation of natural grape must by two yeast strains in bioreactors under aerobic or anaerobic conditions (Y_E/S_ ethanol yield on sugar, Y_A/S_ acetic acid yield on sugar, Y_G/S_ glycerol yield on sugar). Statistically significant differences for each parameter between the [*gar*^-^] and [*GAR*^+^] phenotypes with the same genetic background are indicate by ^∗^ (*p*-value < 0.05) or ^∗∗^ (*p*-value < 0.001). A global view of the experiments is shown in Figures 4, 5 and Supplementary Figures S1, S2.
