# Supplementary figures and images for: Low Phenotypic Penetrance and Technological Impact of Yeast [GAR+] Prion-Like Elements on Winemaking
Source: Front Microbiol. 2019 Jan 9;9:3311. doi: 10.3389/fmicb.2018.03311 (PMC6333647; doi:10.3389/fmicb.2018.03311)

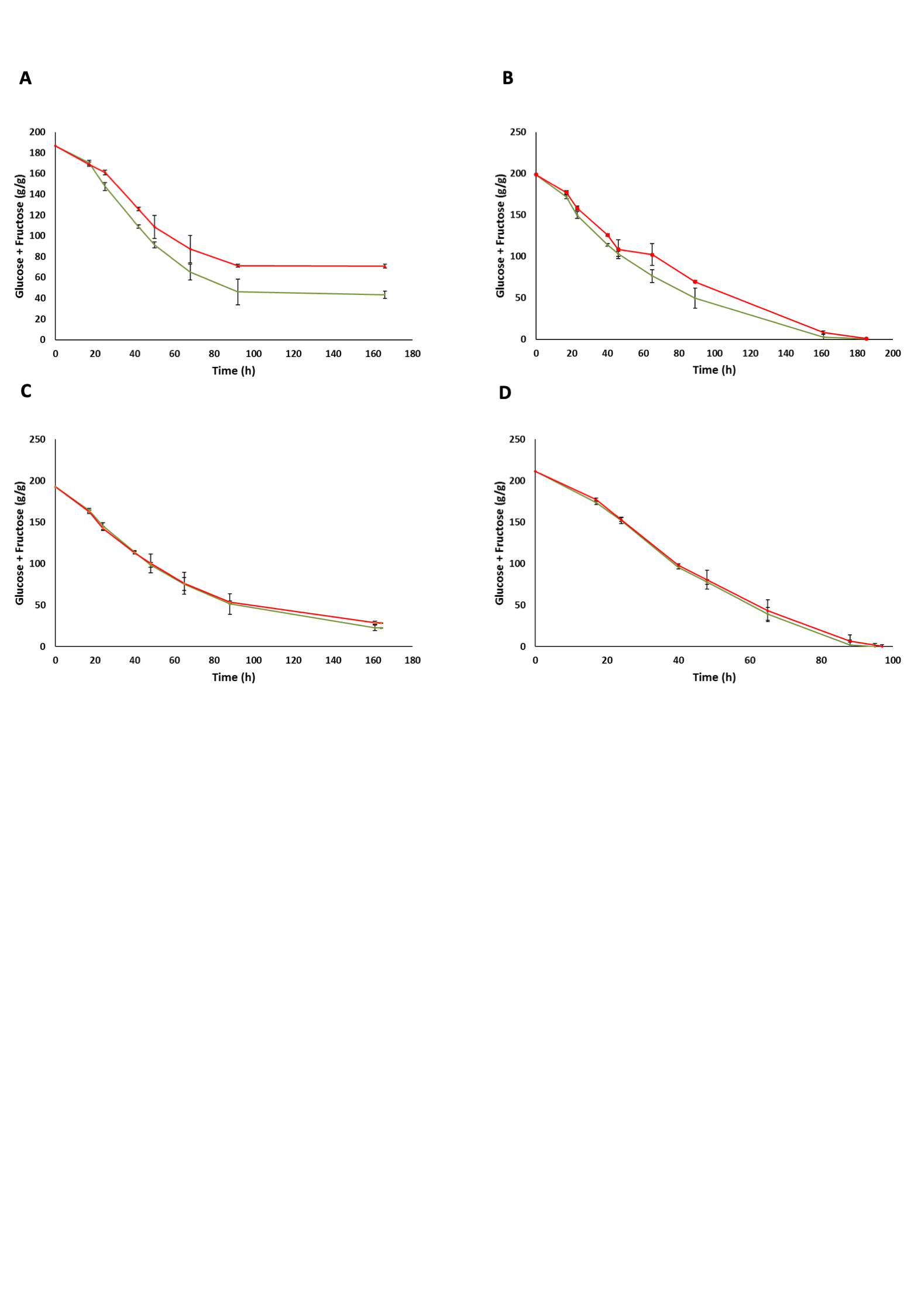

Supplement: FIGURE S1 — Evolution of residual sugar during the fermentation of natural grape must in bioreactors. (A) UCD522 under aerobic conditions. (B) UCD522 under anaerobic conditions. (C) FX10 under aerobic conditions. (D) FX10 under anaerobic conditions. Data for the [gar-] and [GAR+] phenotypes are shown in green and red color, respectively. Error bars indicate ±SD from three biological replicates. [file Image_1.TIFF]

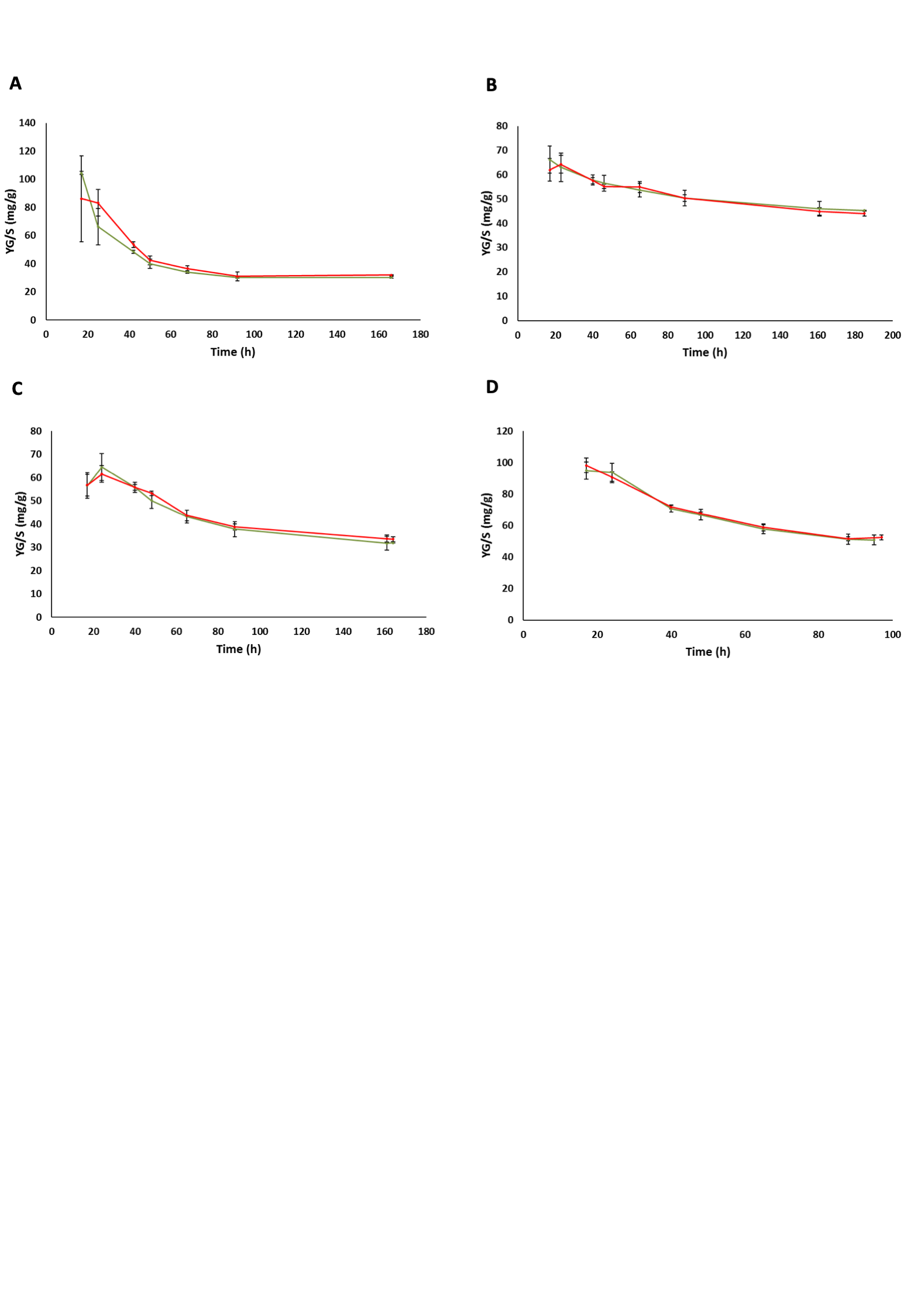

Supplement: FIGURE S2 — Evolution of glycerol yields during the fermentation of natural grape must in bioreactors. (A) UCD522 under aerobic conditions. (B) UCD522 under anaerobic conditions. (C) FX10 under aerobic conditions. (D) FX10 under anaerobic conditions. Data for the [gar-] and [GAR+] phenotypes are shown in green and red color, respectively. Error bars indicate ±SD from three biological replicates. [file Image_2.TIFF]
