# Supplementary material for: Low Phenotypic Penetrance and Technological Impact of Yeast [GAR+] Prion-Like Elements on Winemaking
Source: Front Microbiol. 2019 Jan 9;9:3311. doi: 10.3389/fmicb.2018.03311 (PMC6333647; doi:10.3389/fmicb.2018.03311)
Supplement: TABLE S1 — Final concentrations (10 days) of the main fermentation related metabolites during the fermentation of natural grape must by different yeast strains. [file Table_1.DOCX]

**Table S1.** Final concentrations (10 days) of the main fermentation related metabolites during the fermentation of natural grape must by different yeast strains.

| **Strain** | **Prion state** | **Glucose (g/L)** | **Fructose (g/L)** | **Glycerol (g/L)** | **Acetic acid (g/L)** | **Ethanol (g/L)** |
| --- | --- | --- | --- | --- | --- | --- |
| **EC1118** | [*gar*-] | 0.00±0.00 | 0.37±0.06 | 7.70±0.10 | 0.85±0.04 | 106.99±2.47 |
|  | [*GAR*+] | 0.02±0.03 | 0.63±0.25 | 8.05±0.05 | 0.88±0.02 | 107.79±1.11 |
| **FX10** | [*gar*-] | 0.00±0.00 | 0.32±0.03 | 7.90±0.17 | 1.00±0.05 | 104.99±3.42 |
|  | [*GAR*+] | 0.00±0.00 | 0.40±0.00 | 7.97±0.21 | 0.98±0.01 | 104.57±1.66 |
| **T73** | [*gar*-] | 0.03±0.06 | 4.03±2.03 | 7.27±0.21 | 1.03±0.02 | 102.90±3.34 |
|  | [*GAR*+] | 1.37±0.81 | 17.90±4.08 | 6.23±0.38 | 0.50±0.09 | 96.46±5.53 |
| **UCD522** | [*gar*-] | 0.10±0.17 | 0.30±0.27 | 6.93±0.21 | 0.64±0.00 | 103.11±6.18 |
|  | [*GAR*+] | 0.00±0.00 | 2.90±0.69 | 7.80±0.17 | 0.97±0.01 | 105.65±0.79 |
| **IFI87** | [*gar*-] | 0.77±1.33 | 9.22±0.11 | 7.22±0.08 | 0.71±0.02 | 100.19±6.93 |
|  | [*GAR*+] | 0.10±0.10 | 10.70±2.40 | 7.58±0.20 | 0.53±0.03 | 93.14±5.66 |
| **IFI473** | [*gar*-] | 0.23±0.06 | 2.40±0.31 | 8.75±0.13 | 0.75±0.03 | 98.34±4.02 |
|  | [*GAR*+] | 0.00±0.00 | 1.67±0.21 | 8.27±0.23 | 0.53±0.04 | 103.14±4.02 |
