# Supplementary material for: Low Phenotypic Penetrance and Technological Impact of Yeast [GAR+] Prion-Like Elements on Winemaking
Source: Front Microbiol. 2019 Jan 9;9:3311. doi: 10.3389/fmicb.2018.03311 (PMC6333647; doi:10.3389/fmicb.2018.03311)
Supplement: TABLE S2 — Yield of the main fermentation metabolites and consumed sugars (10 days) during the fermentation of natural grape must by different yeast strains (YE/S ethanol yield on sugar, YA/S acetic acid yield on sugar, YG/S glycerol yield on sugar). Statistically significant differences for each parameter between the [gar-] and [GAR+] with the same genetic background are indicate by ∗ (p-value < 0.05) or ∗∗ (p-value < 0.001). [file Table_2.DOCX]

**Table S2.** Yield of the main fermentation metabolites and consumed sugars (10 days) during the fermentation of natural grape must by different yeast strains (Y_E/S_ ethanol yield on sugar, Y_A/S_ acetic acid yield on sugar, Y_G/S_ glycerol yield on sugar). Statistically significant differences for each parameter between the [gar-] and [GAR+] with the same genetic background are indicate by * (p-value < 0.05) or ** (p-value < 0.001).

| **Strain** | **Prion state** | **Consumed sugars (g/L)** | **Y_G/S_ (mg/g)** | **Y_A/S_ (mg/g)** | **Y_E/S_ (mg/g)** |
| --- | --- | --- | --- | --- | --- |
| **EC1118** | [*gar*-] | 236.78 ± 0.06 | 32.52 ± 0.43* | 3.59 ± 0.16 | 451.84 ± 10.45 |
|  | [*GAR*+] | 236.50 ± 0.28 | 34.04 ± 0.23* | 3.71 ± 0.08 | 455.78 ± 5.16 |
| **FX10** | [*gar*-] | 236.83 ± 0.03* | 33.36 ± 0.73 | 4.24 ± 0.20 | 443.31 ± 14.48 |
|  | [*GAR*+] | 236.75 ± 0.00* | 33.65 ± 0.88 | 4.14 ± 0.04 | 441.68 ± 7.02 |
| **T73** | [*gar*-] | 233.08 ± 2.08* | 31.18 ± 0.92* | 4.42 ± 0.10** | 441.42 ± 11.25 |
|  | [*GAR*+] | 217.88 ± 4.90* | 28.59 ± 1.11* | 2.29 ± 0.40** | 442.57 ± 19.57 |
| **UCD522** | [*gar*-] | 236.75 ± 0.44* | 29.29 ± 0.90* | 2.70 ± 0.01** | 435.53 ± 26.34 |
|  | [*GAR*+] | 234.25 ± 0.69* | 33.30 ± 0.69* | 4.14 ± 0.04** | 451.00 ± 2.54 |
| **IFI87** | [*gar*-] | 227.17 ± 11.88 | 31.82 ± 1.65 | 3.12 ± 0.10** | 440.87 ± 13.48 |
|  | [*GAR*+] | 226.35 ± 2.50 | 33.50 ± 0.61 | 2.34 ± 0.13** | 411.59 ± 26.92 |
| **IFI473** | [*gar*-] | 234.52 ± 0.35* | 37.31 ± 0.62* | 3.19 ± 0.12** | 419.31 ± 16.96 |
|  | [*GAR*+] | 235.48 ± 0.21* | 35.10 ± 0.95* | 2.27 ± 0.16** | 437.97 ± 16.89 |
