# Supplementary material for: Low Phenotypic Penetrance and Technological Impact of Yeast [GAR+] Prion-Like Elements on Winemaking
Source: Front Microbiol. 2019 Jan 9;9:3311. doi: 10.3389/fmicb.2018.03311 (PMC6333647; doi:10.3389/fmicb.2018.03311)
Supplement: TABLE S3 — Final yield of the main fermentation metabolites and consumed sugars after 40 h of fermentation during the fermentation of natural grape must by two yeast strains in bioreactors under aerobic or anaerobic conditions (YE/S ethanol yield on sugar, YA/S acetic acid yield on sugar, YG/S glycerol yield on sugar). Statistically significant differences for each parameter between the [gar-] and [GAR+] phenotypes with the same genetic background are indicate by ∗ (p-value < 0.05) or ∗∗ (p-value < 0.001). A global view of the experiments is shown in Figures 4, 5 and Supplementary Figures S1, S2. [file Table_3.DOCX]

**Table S3.** Final yield of the main fermentation metabolites and consumed sugars after 40 hours of fermentation during the fermentation of natural grape must by two yeast strains in bioreactors under aerobic or anaerobic conditions (Y_E/S_ ethanol yield on sugar, Y_A/S_ acetic acid yield on sugar, Y_G/S_ glycerol yield on sugar). Statistically significant differences for each parameter between the [*gar*^-^] and [*GAR*^+^] phenotypes with the same genetic background are indicate by * (p-value < 0.05) or ** (p-value < 0.001). A global view of the experiments is shown in Figures 4, 5, S1 and S2.

| Strain | Condition | | | | | |
| --- | --- | --- | --- | --- | --- | --- |
|  | Aeration | Prion state | Sugars consumed after 40h (g/L) | YA/S (mg/g) | YG/S (mg/g) | YE/S (mg/g) |
| UCD522 | Aerobiosis | [*gar*^-^] | 77.57 ± 0.91** | 15.19 ± 0.57** | 30.30 ± 0.56* | 275.83 ± 1.93* |
|  |  | [*GAR*^+^] | 60.73 ± 1.72** | 29.42 ± 0.58** | 31.99 ± 0.39* | 264.26 ± 6.20* |
|  | Anaerobiosis | [*gar*^-^] | 84.80 ± 1.70** | 0.37 ± 0.02 | 45.96 ± 3.02 | 358.99 ± 7.32 |
|  |  | [*GAR*^+^] | 73.03 ± 1.60** | 0.30 ± 0.06 | 44.79 ± 1.56 | 365.41 ± 6.30 |
| FX10 | Aerobiosis | [*gar*^-^] | 78.97 ± 1.10 | 8.11 ± 0.09* | 31.94 ± 0.56* | 242.20 ± 2.53 |
|  |  | [*GAR*^+^] | 79.8 ± 1.33 | 10.06 ± 0.66* | 33.50 ± 0.20* | 244.18 ± 9.40 |
|  | Anaerobiosis | [*gar*^-^] | 116.10 ± 0.98* | 0.37 ± 0.04 | 50.80 ± 0.27* | 383.17 ± 0.71 |
|  |  | [*GAR*^+^] | 113.32 ± 0.24* | 0.31 ± 0.05 | 52.49 ± 0.78* | 384.16 ± 9.53 |
